# Embedding Multiperspective Reflections in Co‐Producing a Training Intervention for Care Home Staff: Understanding Group Members' Journeys and Impacts

**DOI:** 10.1111/hex.70739

**Published:** 2026-06-22

**Authors:** Tamara Backhouse, Scarlett Fountain, Peter Davis, Peta Cremer, Anne Francis, Linda Adams, Peter Jackson, Michelle Kiddell, Katie Fray, Gabby Meadows, Revathy Madhav, Anne Killett

**Affiliations:** ^1^ School of Health Sciences University of East Anglia Norwich UK; ^2^ Cavell Court Care Home Care UK Norwich UK

**Keywords:** care home, collaboration, co‐production, power, reflection, training

## Abstract

**Background:**

Co‐production can be used to develop research, resources or interventions that are relevant for the groups they are co‐produced with. We co‐produced a training intervention to improve personal care assistance for people with dementia living in care homes. We aimed to reflect on the process to learn more about the co‐production journeys of all group members and impacts.

**Methods:**

A co‐production group comprised of five care home staff, two relatives of care home residents, two researchers and a public involvement relative/researcher met at the university for 2 h a week, 10 times over an 11‐week period. A care home resident and relative advised the process. We recorded co‐production experiences by each contributing weekly reflections. We used the model of small group development to map out different co‐production journeys.

**Results:**

Care home staff, relatives and researchers had different journeys throughout the co‐production process. Alongside the production of the training intervention, many positive and some negative impacts were identified. Relationships developed, members used and extended existing skills, and new learning, confidence and growth occurred. However, researchers found the process intense, care home staff had to catch up on missed work and one relative felt inhibited at times.

**Conclusion:**

Gaining multiperspective reflections from group members throughout co‐production has produced new learning about how the process can impact people and groups differently. We share our processes, challenges and facilitators which can inform researchers planning co‐production. When co‐producing with care staff, consideration should be given to reduce potential negative impacts on social care settings.

**Patient or Public Contribution:**

The wider study of which the co‐production was part has a lived experience advisory group, hosted by a local care home, who advise 3‐monthly on the design of the study, issues to be addressed, processes of research, documents and findings. The co‐production presented in this article involved five care home staff, two relatives of care home residents and a relative/researcher who is the lived experience lead for the wider study. The co‐production process had two advisors: a care home resident and a relative of a care home resident who met in one‐to‐one meetings with a researcher, their ideas and suggestions and those of a previous stakeholder consultation were fed into the main co‐production group.

## 1 Introduction

Co‐production is increasingly used in research to make sure study processes, outputs or interventions produced are relevant, feasible, and will work for and/or within the communities they have been developed for [1]. Co‐production has multiple definitions but usually refers to service users and carers contributing to the design of and/or delivery of the services they use through partnership working [2, 3]. In research, co‐production is often carried out by working with stakeholders relevant to the study's focus and aims [4] and generally guided by the principles of including all perspectives and skills, sharing power, respecting and valuing knowledge from all parties, reciprocity and building and maintaining relationships [5, 6, 7].

Reported benefits of co‐production in research include making interventions more credible, relevant and feasible for those that will use them [7]. This ultimately increases potential for impact [8, 9]; open creativity and exploration [7, 10], learning of new perspectives and ideas [11], with a move away from typical advisory approaches to the empowerment of co‐producers [10]. Researchers also get an opportunity to engage with, and learn from, those with lived experience relevant to the research focus [11], equally co‐producers can learn more about research and an area that affects them personally [8, 12], while also bringing their individual skillset to this new area [11, 13].

Co‐production can present challenges; it is highly context dependent and requires trust‐building based on the premise of effective communication and respect [9, 11]. Group dynamics can be tricky, for example, agreement or consensus may be difficult to reach, there may be conflict, misunderstandings or confident group members may take over [14]. Managing group dynamics can be difficult, stressful and time consuming for all [15]. Co‐production can be time intensive in other ways too, organising the process, preparation, relationship building and communication all take time, potentially leading to researcher fatigue [8, 11]. There may also be costs to researcher careers due to the work not being valued in academic contexts [16] potentially due to questions regarding validity [11], relying on few voices, or pressure to frame findings or outputs in a certain way [8]. Additionally, other group members may experience inequality in processes [17] and costs to their lives through taking time from their day jobs or usual routines [8]. Research funding is often short term, limiting continued co‐production and building and nurturing of long‐term partnerships and systems [9].

For co‐production to be successful, there is a need for clear and adequate communication of relevant information to enable shared decision‐making [2, 18, 19]. There should be enough resources available to enable co‐production, such as researcher time, flexibility and reimbursement for co‐producers [3, 5, 20]. Stakeholders should be involved from the start, valued, rewarded, included, supported and trained in co‐production principles [3, 18]. The co‐production process should also be reviewed and evaluated to enable continuous learning, to show impact and share processes [3, 7]. To enable genuine co‐production to occur, researchers should adopt ongoing critical reflective practices and communication [21], build trusting relationships [11, 22], be open minded and prepared to learn [23], and be challenged [24].

Care homes provide 24‐h residential housing with support, including meals, assistance with personal care and activities, and some offer qualified nursing care. Care homes are complex settings; they comprise residents' homes and staff members' workplaces. Relatives of residents are also key stakeholders often advocating for the resident they support [25]. When co‐producing with care home communities, several specific barriers and benefits have been identified [25]. Care home staff are busy which creates challenges for their involvement [26] and for the recruitment of residents [27]; additionally, care staff often only provide access to certain residents [28]. There can be fear of voicing opinions or experiences due to potential consequences from other co‐producers [29]. However, facilitators include researchers taking flexible approaches and providing stimulating experiences with benefits for co‐producers involving learning from other co‐producers, valuing different ideas and perspectives [25], and being active participants in social life [30].

The OPTIMISED DEMCARE Study aims to co‐produce a training intervention for care home staff to improve personal care assistance for people with dementia living in care homes and then conduct a feasibility trial. The study adopted Hawkins et al. (2017) [31] framework for co‐producing and prototyping public health interventions. This involved qualitative data collection regarding care home contexts and preferences and experiences of personal care, a stakeholder consultation, co‐production, and prototype testing the intervention in two care homes to find out how acceptable it is for staff before refining it for the feasibility trial, which will determine if it can be implemented and if we can assess it under a trial conditions. This article covers the co‐production processes only, for which, we decided to embed multiperspective reflections throughout.

There is a call to share experiences of co‐production to help others embarking on this journey. Researchers are encouraged to record activities undertaken, who was involved and how, how power was shared, skills developed by co‐producers and desired outcomes [7, 15]. To respond to this, we aimed to reflect on the co‐production process to learn more about co‐production journeys, processes and the impacts of these. This examination of co‐production journeys provides one of the first examples of embedded whole team reflection in co‐production.

## 2 Materials and Methods

### 2.1 Co‐Production

Implementing new practices in care home settings can be challenging [32, 33]. Co‐production was employed as an appropriate method to make sure the training created was relevant and acceptable to those in the settings for which it was developed. To develop the intervention, we drew on the MRC/NIHR new framework for developing and evaluating complex interventions, which emphasises context, programme theory, stakeholder engagement, identifying key uncertainties, refinement of interventions, and economic considerations [34] and the NIHR's Guidance on co‐producing a research project [5]. Co‐production covered the development of the training intervention and not co‐production of the research study.

### 2.2 Recruitment

#### 2.2.1 Co‐Production Group Members

We aimed to recruit people from care home communities: staff, relatives and residents. Researchers gained details of care homes from a Google search, looking for those in close geographical vicinity to the university to enable meetings to take place face‐to‐face with limited travelling for group members. The university was chosen by researchers as the venue for the co‐production meetings to enable group members from different care homes to be together in a neutral environment and to enable candid discussions away from their home, workplace, or relative's home. Eight care homes were contacted via a letter with an enclosed poster and follow‐up telephone call approach. The care homes all specialised in dementia; they represented those providing residential and/or nursing care and a range of sizes up to 80 beds. Two managers responded positively and shared information about the process and role within their care homes. One care home offering dementia and non‐dementia specific residential care with 80 beds had two interested relatives and five interested staff members. No interested people came forward from the second care home. Researchers were keen to involve residents; however, they were reliant on care home staff as gatekeepers sharing information with residents and no residents came forward. A researcher visited those interested, provided a role description and information form, and explained more about the process. The study Lived Experience Lead, a former family carer, also received information. All co‐production group members signed a co‐production agreement form.

#### 2.2.2 Co‐Production Advisors

To ensure further relevance, two people provided advice on the training processes and materials during their development. During the co‐production process, researchers asked staff co‐production group members if they could advertise for residents to be involved within their care home. With permission from a resident, staff introduced a researcher to them to see if they would like to be involved in the co‐production process. Due to already being several weeks into the co‐production process, the resident took on an advisor role. The resident was provided with information and talked through the process with a researcher. They signed an agreement form and took part in five one‐to‐one meetings with researchers at the care home. Researchers were keen to recruit more residents, but at the time of visits, residents were unwell or staff were occupied with their work so unable to facilitate this. Difficulties recruiting residents for co‐production have been noted before [27, 35]. A relative of a resident with dementia at a different care home who had previously advised our research, also advised the co‐production process and took part in five online meetings with a researcher.

### 2.3 Co‐Production Processes

The co‐production group met face‐to‐face at the university 10 times over an 11‐week period. The co‐production sessions ran between 13.30 and 15.30 on Tuesday afternoons with sustenance available throughout (tea, coffee, biscuits, sweets, fruit, cake). Co‐producers were paid the recommended hourly rate for public involvement meetings [36], for 3‐h a week for the 10 weeks to allow time for reflecting, homework and preparation for the two‐hourly meetings. Travel expenses were reimbursed.

### 2.4 Co‐Production Activities

Each co‐producer was given a pack with a notebook, pen and study information. Co‐production took place through:

- presentations of research evidence and stakeholder consultation findings
- ongoing discussions
- creative development of training activities through assessing evidence, thought showers using flip charts or white boards, discussions, generating training materials, review and negotiations
- group work
- testing training activities
- critical appraisals of training activities, decisions, plans and documents

### 2.5 Advisor Activities

Advisors looked at draft training documents and discussed initial ideas for training developed by the co‐production group. They reviewed and commented on facilitator slides, relative posters, training activities, feedback forms and certificates. Their feedback was presented to the co‐production group and incorporated into the training materials.

### 2.6 Reflections

From the second week, each session started by collating reflections on the previous week. A piece of flipchart paper was split into three areas: staff, relatives and researchers. Each group member wrote reflections about the previous week on Post‐It notes and added them to the relevant section. The study Lived Experience Lead had a dual role of researcher and relative and added reflections to each section as they deemed appropriate. Reflections were typed up by researchers and sent around to the group via email with the session notes each week. During the last co‐production meeting each group (relative, staff, researcher) reflected regarding the co‐production processes, what they had learnt, highs, lows, working together with different groups, and general thoughts. Co‐production advisors also reflected on their involvement during and at the end of their five meetings with a researcher taking notes.

### 2.7 Collating and Synthesising Co‐Production Journeys

To examine co‐production journeys, researchers employed a descriptive qualitative content analysis [37]. We collated the reflections in a table, mapping them across the role of the co‐producers and the 10 weeks the reflections referred to, using this as a deductive coding frame. One researcher repeatedly examined the mapped reflections and compared the journeys of the different roles. Journeys were discussed with a further researcher, and an overview was generated. To enable a higher level of abstraction and learning, we deductively used the model of small group development [38] to further map out co‐production journeys for staff, relatives and researchers. The model comprises five stages: (1) forming: group members become acquainted and create ground rules; (2) storming: conflicts can arise as members assert their opinions and group norms are established; (3) norming: the group begins to resolve any conflicts, develops a sense of belonging and works collaboratively; (4) performing: the group achieves optimal functioning and focuses on completing objectives effectively and (5) adjourning: the group goes through a process of separation and reflection. Mapped journeys were written up descriptively.

Co‐production advisors' reflections were collated separately. All co‐production group members and one co‐production advisor are authors of this paper and have read and agree with its content.

### 2.8 Ethical Considerations

The overall study, including the qualitative data collection in a previous stage, was reviewed and given a favourable opinion by the Social Care Research Ethics Committee (24/IEC08/0030; IRAS ID: 330646). To make sure all co‐producers were supported, the research team (two researchers and the Lived Experience Lead) shared their email addresses with the co‐production group and let the members know they could contact any of them at any time. Co‐producer packs also included ‘One moment’ cards to use if co‐producers wanted to speak or pause or slow down proceedings for any reason.

## 3 Results

### 3.1 The Co‐Production Group

The co‐production group comprised of 10 people: five care home staff and two relatives of care home residents from the same care home. The relatives were adult children of residents, they had skills in adult education, drama and participation. Additionally, a former relative (spouse) of a resident from a different care home aligned to the study as public involvement lead helped lead the co‐production with two researchers. The co‐production group were predominantly female 8/10, White British 9/10, with ages ranging from 27 to 67 (mean 48).

Co‐production advisors were one care home resident (female) without dementia but receiving assistance with personal care living in the care home the co‐production group staff worked in, and the two relatives visited but not related to the relatives, and one relative (spouse, female) of a care home resident with dementia living in a different care home.

### 3.2 Co‐Production Processes

We describe our co‐production journeys linked to Tuckman and Jensen's model of small group development [25] (summarised in Table 1). All roles went through the forming, storming, norming, performing and adjourning stages. We then reflect on working together and key outcomes, challenges, and facilitators from the co‐production process. The focus of each of the 10 co‐production sessions and key reflections from co‐production group members are presented in Table 2.

**Table 1:** Co‐production journeys linked to Tuckman and Jensen's model of small group development.

Role	Forming	Storming	Norming	Performing	Adjourning
Staff experience	Excited, nervous, worried	Feeling heard	Valuing teamwork and structure	Valuing teamwork, feeling productive	Feeling proud, sad to end co‐production
Relative experience	Engaged	Trying to understand the process to the goal, learning from staff	Understanding the process, using skills	Enjoying creativity, safety in the group, increased confidence	Achievement, sad to end co‐production
Researcher experience	Balancing sharing enough information with group engagement	Uncertain about sharing power and facilitation skills	Getting in the flow	Trusting the process and group	Relief co‐production was successful, rewarding
Co‐production strategies (preplanned and *unplanned)	Creating shared goals for working together, welcoming input, sharing evidence	Inviting open discussion and critique, *Mixing seating options, *sharing researcher vulnerability	*Introducing structure and weekly aims, *ensuring all members contribute	Using the groups' skills, testing training	Homework, refining materials, *celebration lunch
**←** *Background preparation and organisation* **→**					

*Unplanned co‐production strategies.

**Table 2:** Co‐production sessions and reflections.

Week	Session content	Staff reflections summary	Relative reflections summary	Researcher reflections summary
1	Getting to know one another Why are we here? What is co‐production? What is our co‐production goal? How will we work together? The research evidence	*Reflections on process* Nervous/worried about contributing Excited to start a new journey! Want to make a tiny difference. Informative Welcoming Good communication *Learning* Enjoying learning I was reminded about the complex, nuanced nature of care work—being responsive to the whole person.	*Reflections on process* Exciting! Good to get started. Was bored for the first slide, then became engaged and enjoyed it. Enjoying the mixture (of staff/relatives/researchers) Good idea to include care staff. *Learning* Realised I'm not always flexible with Mum.	*Reflections on process* Happy/relieved to have such a supportive and keen group. Ready to get stuck in! This is a group process—need to mix the group up so relatives and care staff are mixed and not separate to one another. Need to make things more equal—too much standing at the front—slides—leading—teacher style. —afraid of losing people Hard to get people up to speed and make it exciting/participatory.
2	Getting to know one another Reflections of last week's session Care home staff member's poem OPTIMISED‐DEMCARE training logic model Stakeholder consultation summary Intervention content discussion Aligning with policy and industry	*Reflections on process* Felt treated equal. Everyone gets the chance to share their opinion and experience. Different perspectives of different people Looking forward—working together. Confidence to speak up our views and suggestions. *Learning* Staff need to think of themselves and take care of themselves.	*Reflections on process* Good to feel more confident speaking as a group—sharing ideas. Would be good to feel clearer about how to shape training. *Learning* Resident choice trumps relative's wishes.	*Reflections on process* Off topic discussions. Need to structure more. Worried we are not getting enough done in the time. Group appeared more cohesive, integrated, and chattier. Feeling a little out of control. Some decisions made— yay! Am I facilitating clearly and/or strongly enough? Am I a good facilitator?
3	Reflections of last week's session Ways to make changes at the care home level Local needs assessment—what will we ask care homes? Formal commitment—what are homes agreeing to? DEMCARE Champions? Role and characteristics Open discussion Quick reflections of structured session	*Reflections on process* Session went very well. More structured. Work well as a team.	*Reflections on process* Started to process information. Feeling more sure of the process now. Good to have the stakeholder consultation as basis for discussion now. *Learning* Realised I'm not always flexible with my mum.	*Reflections on process* Felt a little teacher‐ish—is the power balance equal? Made lots of progress today—yay! Nice to get onto the core business of developing the training. Felt like the team spoke up more. Great involvement from the team. The groups are so knowledgeable, enthusiastic and responsive. Need to make sure everyone has a chance to speak/take part as much as they would like. Stakeholder opinions versus co‐production group opinions—who has the final say? —enough ownership for the co‐production group? Lots of good discussions. Structure worked well and was productive. More relaxed about making progress.
4	Reflections of last week's session Relative‐led session—how the brain works. Timing of training Training content Whole group developed one training activity. Considering options for focus/activities Split into two groups and developed two training activities	*Reflections on process* Great to work in teams to create activities. Empathy. Teamwork. Understanding. Structured activity.	*Reflections on process* It was great to start with the talk from relative—made us think differently. Enjoyed the session. Feel like it's coming together. Felt constructive to split and focus on the training activities. *Learning* Positive words	*Reflections on process* Great to get the content refined! Separate groups worked well. Great activities developed. Relative's slot was amazing! Good teamwork. Got lots done. Are we covering all we need to? Really good engagement from the group. Felt a bit ‘out of it’ with the break last week. Great session. Lovely to have a relative do a session. *Learning* Brain networks.
5	Reflections of last week's session Split into two groups and developed two training activities. Whole group developed one training activity. Tried one activity out between us	*Reflections on process* Constructive session. Activities are coming along well. Team is working great. Yummy cake and great singing. Things are coming together. *Learning* Felt thoughtful when discussed ‘if I was you?’ Understanding of emotions.	*Reflections on process* Fun session. The training is taking shape. Positive to work through the tasks together—ideas sharing.	*Reflections on process* Drawing on the group's skills. Great teamwork. More bonding. Great to get training content created. Need to practice the activities? Another good week of creating training content. The group has so many skills and experiences they are bringing to this. Group was excellent. Lovely to find out about the group more—trying the activity.
6	We tried out all six training content activities Re‐developed one of them	*Reflections on process* Activity was good and came together really well. And revamped one better. Great teamwork. Sessions are going well. Good teamwork.	*Reflections on process* Getting increasingly comfortable to share ideas, explore amendments and change things. Felt very real working through the activities, great to experience them.	*Reflections on process* Nervous to test/lead the activities! Am I facilitating activities well? Good idea to recreate one training method and glad the team spoke up. Nice to tweak or change the activities to make them better. We have made so much progress. Percy Pigs (sweets) went down well. Four more weeks to go—eeek! But we are getting there! Feel like the content is coming together. Great week again creating and refining training content. Great to test the activities. Dynamic group work. Nice to get candid thoughts.
7	Reflections of last week's session Supporting practice change Training content— additional activity created to match evidence we had not covered. Homework to look through training activity pack	*Reflections on process* Teamwork as always. Great teamwork. Everything coming together. Feeling proud of the whole thing. Great to come up with activities together. The training is coming together. Pride. Safe. More ideas. More suggestions and alterations as a result of teamwork.	*Reflections on process* Great team. Started off tired and perked up. Enjoyed the creativity. Starting to really be able to visualise the training and impact.	*Reflections on process* Feeling a bit teacher‐like again. Nicer in this room. Did I make too many decisions or steer the group too much? First homework given. Lovely to have a relative take on the extra activity organisation— the new activity is such a good addition to the training! We got through so much stuff today! Concerned this week was less exciting than the previous three. Good decisions made balancing feasibility/practicalities and what we need as trainer co‐producers/researchers. Weighing up pros and cons of ideas. Was I too prescriptive? Nice to be critical and not choose every option. Detail versus overview people. Liked sitting in a group/circle. The group whizzed through the content today! Felt like I kept interrupting too much—researcher role? Did feel a bit like teacher and student roles with the content on the board—is there an alternative to show the team necessary info? Printing is not very environment friendly. Felt like everyone was honest and straightforward with decision making. Feels like the training is coming together and it's good to enable the group to review the materials for the facilitator.
8	Reflections of last week's session Homework review Content development: Slides for the DEMCARE Training facilitator planned. Lesson plan and training order Relative information Manager needs assessment and information. Homework— look through new training documents	*Reflections on process* Good teamwork. Good decisions being made about each session. Teamwork as always.	*Reflections on process* Relative info sheet—was good to discuss this. Feel confident and heard.	*Reflections on process* Still feeling a bit teacher‐like again So much to get through—a lot of admin/detailed items Very nearly there now Great to see a relative at the whiteboard. Loved my Birthday Cake! Really tweaking the training now—smoothing the edges Have we covered everything? What have we missed? Interesting to get insights from the relatives. Have the group had enough control—say in everything? Lovely idea to celebrate with lunch during the last session. Lots covered
9	Reflections of last week's session Homework review Review of: Slides for the DEMCARE training Facilitator. Relative information Logic model/theory of change updates Manager needs assessment and information. DEMCARE Champion information Homework— look through toolkit	*Reflections on process* More discussion on the structure of training. Good teamwork as always. Getting to the conclusion. Sad for it to be coming to an end.	*Reflections on process* Sense of achievement. Bit sad to be finishing.	*Reflections on process* Long, tiring session of reviewing documents. Teacher—student set up but balancing printing/environment versus active involvement. Hay fever day! Great input from the team! Team decisions versus my instinct to put in more details—team keeping me on track. Nearly there with all training components. More homework—aware of burden on co‐producers but need input—balance tricky—co‐production versus researchers doing actions due to time/potential burden. Co‐production has been such a success! Celebrating a relative's success—job. Have we thought of everything we need to? Felt a very tiring session. Technology issues again! The team has a great input and is not afraid to say what they think. Feels amazing that co‐production has been very successful. Feels rewarding to think that as a team we have created the training content. Relative's ginger biscuits Got lots of input. Great group.
10	Group lunch Reflections of last week's session Homework review Reflecting on the whole co‐production process Thank you from all to all. Next steps Goodbyes	*Learnt* Point of view of family/researchers. Flexible. Increased confidence—better carers than when we first stepped in the room. Problem solving. Developed emotional intelligence. How to carry out training or create one. *Highs* Good teamwork. To share experiences. Getting to know new people. Cake. Lunch. Sweets. I feel very proud to of been part of it. *Lows* Low—phot**os!** *General* DEMCARE champion/hopefully has time given to do everything. Good teamwork. Sessions have been organised and productive. Good communication. I think the whole project has been fun and very productive. I think what we have done is fantastic and will help care homes across the country. I hope it is accepted and appreciated by the stakeholders. Everyone in that room has worked hard and come up with great ideas. Have to catch up with work when return to the care home after co‐production meetings. At the beginning, I had no idea how we would create the training. *Working together with relatives of the residents you care for* Strengthened our relationships with them. Seen their point of view. Got to know them better as individuals. It will help improve their relative's care.	*Learnt* Working as a team. Reinforced that my dad's in good hands. Tuesday's will not be so much fun. Staff issues that I was unaware of re. barriers and management, and so forth. I can still do it. I have learnt more about dementia care. I feel that I have more understanding of carer's roles and responsibilities and the extent of the challenges they face. I have developed more understanding of how to co‐create —how to input ideas and shape a piece of work collectively. I learnt that it is an imperfect sector with such big challenges around recruitment, finance and strategic management—and that it could be frustrating to try and tackle these in discussion. It was not the forum for that. I gained more understanding of the process, of personal care, of the research and of DEMCARE. *Highs* Meeting new people. See the confidence of participants increase. Creativity and fun to be a part of something that will make a difference. 5 min training. Paying for my November retreat. It was great working in the team—we got along so well, and a positive, inclusive and safe environment was created by researchers. It felt rewarding, informative, impactful. Like there would be a legacy from the training which would be very important to the development of quality dementia care. *Lows* No lows for me. *General* Positivity of all, with enthusiasm. Well led. Everyone contributed. Humour. Got more and more safe. Confidence raising. Felt free to be honest. Made friends. Excellent facilitation— really lovely encouraging atmosphere. A great opportunity to connect with people. There was a lot of work that had been done already on the research project—I understood as we went along that we were fine tuning, shaping. *Working together with staff who look after your loved ones?* Appreciation of their work. Openness to develop,and so forth Raised awareness of staff issues. Respect for their experience and openness to develop skills. I might have felt more able to be open if the work was with a different care home.	*Learnt* Okay to relax/let it happen. Preparation is key—organising—keeping up with processes. Got to get info across—teacher/student necessary sometimes. Flexible/organic. About others. Paparazzi pics. *Highs* Relationships—getting to know one another. Nicknames. Laughs. Cakes—birthdays. Creating the training. Getting the job done. Involvement. *Lows* Balance—directing/laugh/keeping on track. Very hot room. Allergy central. Keeping on the train. *General* Special occasions. Great team. Fun. Pleased with the training we've developed. Uncertainty—has everyone had a say? Have we covered everything? Researcher role—input/view—throughout—evidence—but too much? Gone quickly/ One moment card—not used! Mobile numbers—not used! *Experience of working with care staff and relatives* Care Staff: Learnt about care home processes. Acknowledging their knowledge. Rely on their views. Relatives: Good to get personal aspects. Hearing stories/perceptions. Overall: Apologies—communication was good. Payment. Sustenance.

### 3.3 Forming: Week 1

**Figure 1:**
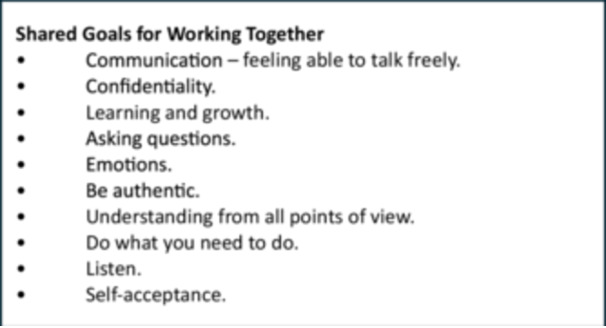
Co‐production shared goals for working together.

During the first meeting, the group got to know one another and created shared goals for working together (see Figure 1). These were agreed and reflected the group's aim to make co‐production a safe and productive space to work together.

Staff started off excited about the prospect of working together and making a difference, but also worried and nervous about contributing and co‐producing the training intervention. They found the meeting welcoming. Staff enjoyed learning, found the initial study information and evidence informative. One staff member was reminded about the complex nature of dementia care work and thinking of the whole person.

Relatives initially found the co‐production process exciting and felt it was good to get started. They enjoyed the mixture of roles in the group and thought it was a good idea to include care staff. Relatives reflected on realisations about their relationship with the person with dementia they supported, learning from co‐production discussions that they could be more flexible.

Researchers were happy and relieved to have such a supportive and keen group and were ready to get started. Researchers found it unsettling at first to share power and lose overall control over what the intervention would become. Initial reflections included uncertainty whether the co‐production group would find the process positive and engaging. Researchers also had concerns about balancing providing enough information to upskill co‐producers to make informed decisions without creating a teacher/student situation with unequal power, making the process non‐participatory, or losing people. Researchers worked hard to share power and create a cohesive group by offering all a chance to contribute and openly inviting critique or disagreement. Researchers reflected after the first meeting that they needed to mix the group up so relatives, care staff and researchers were not sitting separate to one another. Consequently, during the next meeting they sat themselves separately to split up the seating options.

### 3.4 Storming: Week 2

The storming stage progressed quickly, without major conflict, and was mostly identified in Week 2 where multiple issues surrounding care of people with dementia were discussed from all perspectives. Staff stated that they felt treated as equals, with everyone getting the chance to share their opinion and experience. They valued feeling heard, hearing different perspectives from different people and quickly gained confidence to speak up with their views and suggestions. Staff were reminded that they needed to take care of themselves as well as the residents.

Relatives were trying to understand the process to the goal and felt unclear about how to shape the training. One relative reflected that it was good to feel more confident speaking as a group and sharing ideas. Relatives also learnt more about dementia and that resident choice in the moment is prioritised by care staff over relatives' wishes, which made them reassess how things worked in care homes and their role regarding their loved one's care. They gained more understanding of the care home sector and the challenges regarding recruitment, finance and strategic management and reflected at the end of the process that it could be frustrating to try and tackle these in discussion. They learnt more about carer's roles and responsibilities and the extent of the challenges they face.

The second meeting included lots of discussions which enabled the group to bond and become more integrated; however, researchers reflected that some conversations were off topic, and they were concerned there was not enough progress. One researcher reflected that they felt a bit out of control and questioned whether they were a good facilitator and whether they were facilitating clearly enough. As all reflections were shared with the whole group each week post‐meeting, a relative later reflected that this researcher vulnerability was refreshing to see.

### 3.5 Norming: Weeks 3 and 4

The norming stage did not take the group long to get to and was seen mostly in meetings three and four where we started to create the training. Researchers introduced more structure to meetings, with clear aims for each meeting, this was well received by the wider group and led to more progress, which reassured researchers. Staff reflections turned to valuing the new structure and teamwork; how the group was working together well and had effective communication and understanding. The training was underpinned by the theory of emotional intelligence for staff. During this stage, due to incorporating activities related to emotional intelligence into the training, staff increased their understanding of emotions and further developed their own emotional intelligence.

Relatives reflected they had started to process the information and felt surer of the co‐production process as we were becoming more productive. One relative reflected on the value of having the stakeholder consultation (a previous stage of this study [39]) as basis for discussion now. During this stage, a relative offered to start the meeting with a 5‐min session on how the brain works and the importance of reframing our thinking by using energising and positive words. Reframing would be useful to incorporate into the training; for facilitators of the training to reframe issues for staff in training sessions and for trainee staff to reframe situations with residents during personal care interactions. This session was well received, valued and revered by the whole group and demonstrates underlying empowerment—relatives were using their existing skills for the good of the group. Relatives reflected that it was constructive to split into mixed groups to focus on different activities and that it was good to see the training come together.

Researchers reflected on the positive engagement from the group, use of individual's skills and experiences, bonding, and good teamwork. They highlighted the need to make sure everyone had a chance to speak and take part as much as they would like. Therefore, in subsequent meetings everyone was encouraged to speak, write and lead as much as they wanted to. Researchers reflected on the role of the stakeholder consultation evidence versus the co‐production group ideas questioning who has the final say and whether there was enough ownership for the co‐production group. Researchers were becoming more reassured that progress was being made.

### 3.6 Performing: Weeks 5–8

During the performing stage we were generating and refining most of the training content. We drew heavily on the co‐production groups' existing skills related to dementia care, care home staff training, adult education, participation and drama. We tested out the training activities, one of which involved sharing information about what we do for self‐care or to nourish ourselves, this activity meant we learnt a bit more about each other and this made us bond further as a group. The progress of creating and testing the training was highlighted by staff, who reflected that sessions were going well; work was constructive, and everything was coming together. Teamwork was valued, and the group was working cohesively. Some staff were starting to feel proud of the training and progress. The creativity and ability to revamp and alter training activities due to suggestions were valued.

As meetings progressed, relatives reflected that they enjoyed the creativity, felt confident and heard, and were increasingly comfortable to share ideas, explore amendments and change things. Relatives also valued teamwork, reflecting that it was positive to work through the tasks together. Relatives enjoyed discussing and creating the relative information poster, and working through the activities and experiencing them, which made them able to visualise the training and potential impact. Additionally, they professed developing more understanding of personal care, of the research, and how to co‐produce, input ideas and shape a piece of work collectively. One relative reflected that they came to understand that we were fine tuning, shaping previous work through co‐production.

During this stage, researchers relaxed a bit and allowed the co‐production process to take shape. However, researchers reflected on their concerns throughout: Were they covering everything they needed to? Were they steering the group too much? Did the group have enough control? Were the meetings exciting/engaging? For example, researchers still felt like teachers, bringing the group back to the evidence and previous stakeholder consultation and shaping the activities around this, meaning that co‐production was free within the constraints of the evidence, not totally free.

Researchers were pleased that the group was candid, critical and not afraid to say what they thought. For example, when testing the training activities, one was discarded and recreated due to being too taxing and dull. Researchers also reflected positively on relatives taking the lead by writing on the whiteboard and organising a new activity. Researchers learnt that it was okay to let co‐production happen, that they could relax, but that preparation was key.

### 3.7 Adjourning: Weeks 8–10

In the last few weeks, staff reflected they felt proud of the training and co‐production and hoped the training would make a difference. One staff member reflected feeling safe and another that everyone had worked hard and had great ideas. Staff reflected that they were sad for the process to be ending. They highlighted the problem solving, thought‐provoking and flexible nature of co‐production, and that they had increased in confidence over the co‐production period. With one staff member stating at the end, that they were better carers than when they first stepped in the room. Learning included being more aware of relatives' and researchers' point of views, finding out how to conduct or create training and the need for care home staff to take care of themselves. Reflecting on the whole process, staff valued the good teamwork, sharing of experiences, the fun, getting to know new people and the sustenance. Staff also reflected that after co‐production meetings they had to go back to their shift and catch up on work missed while they were away from the care home.

Near the end, relatives reflected on the humour and fun that occurred. Generally, relatives found co‐production a great opportunity to connect with people and made friends. One reflected that they had renewed confidence, they can still contribute and have valuable skills to use. Relatives also reflected that they had a sense of achievement and felt a bit sad to be finishing. One relative indicated that Tuesdays would not be so much fun after co‐production ended and shared that they would be going back to their usual routines.

Relatives had enjoyed working in the team and seeing the confidence of group members increase. One reflected that meetings were well‐led with excellent facilitation and there had been a positive, inclusive encouraging atmosphere and safe environment created by researchers. Relatives liked the creativity and found it fun to be a part of something that would make a difference, stating it felt rewarding, informative and impactful.

In the last stages, training documents needed to be reviewed by the group, and researchers found it difficult to balance the need for printing against using the overhead projector presenting content from the front of the room in a teacher/student format for discussion and for editing. Co‐producers were given documents to review for homework and researchers reflected on balancing the burden on co‐producers against needing their input. One researcher reflected that the group decisions to keep documents brief went against their instinct to add in more details to the training package.

Final researcher reflections showed relief that they believed co‐production had been a success, the team had been committed and enthusiastic, and that it felt rewarding to have created the training together as a team. High points included the relationship building and getting to know one another, birthday celebrations, celebratory lunch at the last meeting, a relative bringing in homemade bakes, the nicknames that were created in the last meetings when the group was so sure of each other, the laughing and co‐producing the training. Keeping the co‐production going had been intense for researchers with preparation, organisation, facilitation and post‐meeting work each week for the next week.

### 3.8 Working Together: Staff, Relatives, Researchers

Staff found working with relatives of the residents they cared for valuable for strengthening relationships, getting to know the relatives better as individuals, and seeing their point of view. Some thought it would help improve the residents' care due to knowing more about them. Relatives had new appreciation and respect for staff's work and the issues they faced. They were impressed with the openness of staff to develop through the co‐production process. However, one relative was slightly inhibited and reflected that they might have felt more able to be open if co‐production was with staff from a different care home. Researchers found working with care home staff useful for learning about care home processes, they recognised care staffs' knowledge and could rely on their views and skills. Collaborating with relatives had enabled key skills in creative methods to be drawn on, stories to be heard and personal aspects to be considered regarding relatives and residents' experiences.

### 3.9 Co‐Production Advisor Reflections

Advisor reflections covered appreciation of being able to voice their thoughts, be involved in decisions, and help those with dementia and care staff. They found it empowering to speak up from their experiences. Advisors stated they looked forward to meetings and had developed a friendship with the researcher. The relative reflected that they had learnt a lot from the content discussed. The resident reflected that it felt right to be consulted and they were happy to give information on topics to improve care.

### 3.10 Co‐Production Outcomes, Challenges and Facilitators

Table 3 presents the key outcomes, challenges and facilitators of our co‐production. Co‐production also led to the care home drawing on the experience to enter a regional award and becoming finalists for the collaboration in social care award, and a relative, inspired by using their skills in co‐production, signed up for a course. Friendships were created and deepened, and there are ongoing collaborations between researchers and the care home, with the care home resident still advising the wider study.

**Table 3:** Key outcomes, challenges and facilitators of co‐production.

Outcomes	Challenges	Facilitators
Training intervention and processes Increased confidence Learning Skill acquisition and renewals Friendships created and/or deepened Care home as an award finalist	Sharing enough knowledge to upskill co‐producers to make informed decisions without losing their interest or creating marked power imbalances Gaining authentic input without creating burden Getting to grips with research/co‐production processes and aims (staff and relatives) Being candid with those from the same social care setting Keeping up with the intense process (researchers) Reducing impact on social care settings	Co‐producing shared goals for working together early on Being clear about the aim of the co‐production from pre‐recruitment throughout Co‐producers feeling supported and willing to input Researchers showing vulnerability and openly welcoming critique and challenge Adapting the structure of sessions (to make sure progress was achieved and all group members had input) Celebrating successes and occasions together Sharing information about ourselves as part of testing a training activity Utilising co‐producers’ existing skills

## 4 Discussion

We co‐produced a training intervention for care home staff to improve personal care assistance for people with dementia. All group members reflected on the co‐production process throughout, enabling the co‐production journeys of staff, relatives and researchers to be known. Findings are novel in that they show all roles went through Tuckman and Jensen's stages of small group development—forming, storming, norming, performing and adjourning—in slightly different ways as they engaged with the co‐production process. Ultimately co‐production was successful; reflections were largely positive, a dynamic training intervention was co‐produced, and challenges and facilitators recognised. Impacts included co‐producers gaining new learning, using or extending existing skills, growing in confidence and deepening relationships. However, there were implications for involving co‐producers from the same setting and care staff taking time away from the care home.

Our co‐production reflections endorse existing literature, showing the benefits of co‐production. Improvements in skills and confidence have been noted from other co‐production processes [13, 40], as have learning and knowledge acquisition [8], empowerment of co‐producers [41] and co‐producers using their own skillsets [13]. Our reflections build from other literature highlighting the relational benefits from co‐producing [42, 43], showing the impact of working with people from different roles from within the same social care setting. Relatives and staff learnt about each other, grew deeper bonds, which strengthened their relationships. This potentially improved care for relatives' family members within the care home as staff knew more about them. This potential positive impact needs exploring in future research. Conversely, involving staff and relatives from the same care home created inhibition for at least one co‐producer and maybe others; fear of voicing opinions due to other co‐producers has been identified before [29]. Researchers should consider the impacts of the group dynamics when recruiting co‐production group members and planning co‐production work, and work with co‐producers to overcome any not addressed.

Our reflections build on existing evidence relating to creating co‐production as a safe space [9, 13, 15]. During our co‐production, trust was built between group members in multiple ways including researchers reflecting openly on their own potential shortcomings. This shared vulnerability may have been crucial in deepening connections and creating a safe space for all to reflect [44]. However, there could have been an emotional toll for researchers [45] or a risk to researcher credibility within the group. In their turn, staff were open and honest in their opinions, and relatives asked questions, shared experiences and skills, and were candid with their thoughts. The safe space created was reliant on all parties being prepared to work toward the common goal with sensitivity, which they did, and on researcher interpersonal skills, which have been noted as undervalued in research [46].

Related to trust and safety is sharing power, which can be difficult in co‐production [47]. Our reflections add to knowledge by showing multiple ways of how power sharing was enacted. One way researchers tried to share power was by passing on knowledge of the evidence base to enable group members to make informed decisions. However, this very act created unequal dynamics in meetings where researchers had to present information or provide printed text to the group to read. Group members, including researchers, may have expected equality and shared power but found they were being guided by researchers and/or evidence [48]. However, the aim was to create an evidence‐based intervention, so researchers tried to manage expectations, highlighting the job of co‐producers was to use the evidence to create training.

Co‐production group members were highly skilled, relatives had skills in drama, participation and adult education, staff had skills in dementia care, poetry, art and training staff members, and researchers had skills in research and in working sensitively with stakeholders. Together these skills were utilised [13] to create a dynamic and participatory training intervention for care home staff. By welcoming and using co‐production group members' skills, we were able to share power further as everyone had something to offer the process and made valuable contributions [42]. Co‐production can be tokenistic [49]; however, in our group, staff and relatives inputted greatly, often leading the creativity in activities and forming ideas for training activities or highlighting key problems with what we had co‐produced. Co‐production necessitates that researchers are dependent on group members' input [48], and the group did not disappoint. Power sharing was everyone's responsibility as group members had to be willing to take ownership and contribute.

Researchers found that the co‐production journey intense, keeping up with the preparation, thinking and work between sessions to keep progress on track had been taxing. Co‐production intensity has been discussed before in relation to reducing value and researcher fatigue [8, 50]. In our process, the co‐production schedule increased intensity through creating considerable required actions between sessions for researchers [50], which enabled momentum to be maintained during, yet prompted researchers to reflect on their relief in the adjourning stage. Ten 2‐h sessions had been the right amount for the co‐production work needed to develop the training.

## 5 Strengths and Limitations

We co‐produced training with an intense 10‐week commitment from all co‐production group members. The process drew on co‐production guidance and researchers tried hard to enact co‐production principles. An indicator of the success of the co‐production process is that the co‐developed training, including its form and activities, are vastly different than they would be if the researchers had developed them alone. However, co‐production was limited by not including people with dementia and only having one care home resident involved. Our stakeholder consultation [39] which informed the co‐production process had included care home residents and a person with dementia. Nonetheless, a learning point would be to spend more time building relationships with care homes and working with them as gatekeepers to enable resident recruitment to the co‐production process early on. On reflection, hosting co‐production meetings at care homes and having shorter meetings could make it easier for residents to take part. However, locating meetings within care homes could also make it more difficult for staff group members, who may be called away to other duties during co‐production.

Staff and relatives were from the same care home, although this enabled deeper bonds to be created, this may have also hampered some discussions. It could also be possible that co‐production group members were inhibited from providing negative reflections due to researchers reading them or the knowledge that they would be shared weekly with the whole group. Researchers conducted the synthesis, which could have limited further insights from co‐production group members and biased the findings. Additionally, for this article, we synthesised the reflections of groups together which could mean individual journeys were different from others in each group.

## 6 Implications for Researchers

Care staff briefly reflected that they had to catch up with work missed when they returned to their shift after the co‐production meetings. This was due to the care home manager releasing some staff off shift to attend, which researchers were initially unaware of. This practice could generate extra burden on staff, and the care home, for partaking in co‐production. This example shows co‐production in the social care sector can be tricky and wider implications of co‐production may not be fully known. Research burden on care homes has been highlighted before [51]. Researchers planning future co‐production within the social care sector should work to reduce potential wider burdens, particularly checking that staff involvement will be backfilled.

Learning from our co‐production processes, challenges and facilitators can be used by other researchers embarking on a co‐production journey. Researchers should also carefully consider how knowledge will be shared, burden on co‐producers will be reduced and co‐production background processes will be fulfilled in between sessions.

Co‐producers in care home research are not commonly involved in an evaluation of the process [25]. We found reflecting on co‐production processes to be a valuable method of evaluating our co‐production process and has provided descriptive knowledge of how co‐production journeys can differ from role to role; however, formal methodologies such as appreciative enquiry can be used provide theory‐based evidence [52]. Researchers should consider how they may reflect on and evaluate their processes with co‐producers before undertaking co‐production [20].

## 7 Conclusions

We elicited reflections from each co‐production group member each week throughout the process of co‐producing a training intervention for care home staff. This is one of the first examples of embedded multiperspective reflection. The value of the embedded reflection in learning about differential experiences and impacts of the process is demonstrated. In this case, our reflective process enabled learning about the experiences, processes, challenges and facilitators of co‐production. When co‐producing with care home communities, consideration should be given to staff burden, reducing potential negative impacts on social care settings, and complexities of involving co‐producers from the same settings; however, the benefits for those involved may outweigh any negatives.

## Author Contributions

**Tamara Backhouse:** conceptualisation, funding acquisition, investigation, methodology, visualisation, supervision, writing – original draft, writing – review and editing. **Scarlett Fountain:** investigation, methodology, writing – review and editing, project administration. **Peter Davis:** investigation, writing – review and editing. **Peta Cremer:** investigation, writing – review and editing. **Anne Francis:** investigation, writing – review and editing. **Linda Adams:** investigation, writing – review and editing. **Peter Jackson:** investigation, writing – review and editing. **Michelle Kiddell:** investigation, writing – review and editing. **Katie Fray:** investigation, writing – review and editing. **Gabby Meadows:** investigation; writing – review and editing. **Revathy Madhav:** investigation; writing – review and editing. **Anne Killett:** conceptualisation, supervision, writing – review and editing.

## Conflicts of Interest

The authors declare no conflicts of interest.

## Data Availability

The authors confirm that the reflections supporting the findings relating to the co‐production journeys are available within the article.
